# The Effect of Endolymphatic Hydrops and Mannitol Dehydration Treatment on Guinea Pigs

**DOI:** 10.3389/fncel.2022.836093

**Published:** 2022-04-11

**Authors:** Shu-Qi Wang, Chen-Long Li, Jing-Qi Xu, Li-Li Chen, You-Zhou Xie, Pei-Dong Dai, Liu-Jie Ren, Wen-Juan Yao, Tian-Yu Zhang

**Affiliations:** ^1^Department of Facial Plastic Reconstruction Surgery, Eye and ENT Hospital of Fudan University, Shanghai, China; ^2^ENT Institute, Eye and ENT Hospital of Fudan University, Shanghai, China; ^3^Hearing Medicine Key Laboratory, National Health Commission of China, Shanghai, China; ^4^School of Mechanics and Engineering Science, Shanghai University, Shanghai, China; ^5^Shanghai Institute of Applied Mathematics and Mechanics, Shanghai, China

**Keywords:** endolymphatic hydrops, dehydration therapy, Ménière’s disease, laser Doppler vibrometry, cochlear impedance

## Abstract

**Background:**

Endolymphatic hydrops (EH) is considered as the pathological correlate of Menière’s disease (MD) and cause of hearing loss. The mechanism of EH, remaining unrevealed, poses challenges for formalized clinical trials.

**Objective:**

This study aims to investigate the development of hearing loss, as well as the effect of dehydration treatment on EH animal models.

**Methods:**

In this study, different severity EH animal models were created. The laser Doppler vibrometer (LDV) and auditory brainstem responses (ABR) were used to study the effects of EH and the dehydration effects of mannitol. The LDV was used to measure the vibration of the round window membrane (RWM) reflecting the changes in inner ear impedance. ABR was used to evaluate the hearing changes. Furthermore, tissue section and scanning electron microscopy (SEM) observations were used to analyze the anatomical change to the cochlea and outer hair cells.

**Results:**

The RWM vibrations decreased with the severity of EH, indicating an increase in the cochlear impedance. The dehydration therapy lowered the impedance to restore acoustic transduction in EH 10- and 20-day animal models. Simultaneously, the ABR thresholds increased in EH models and were restored after dehydration. Moreover, a difference in the hearing was found between ABR and LDV results in severe EH animal models, and the dehydration therapy was less effective, indicating a sensorineural hearing loss (SNHL).

**Conclusion:**

Endolymphatic hydrops causes hearing loss by increasing the cochlear impedance in all tested groups, and mannitol dehydration is an effective therapy to restore hearing. However, SNHL occurs for the EH 30-day animal models, limiting the effectiveness of dehydration. Our results suggest the use of dehydrating agents in the early stage of EH.

## Highlights

- This study investigated the development of hearing loss on guinea pig EH models, as well as the effect of dehydration treatment. By combining analogical observations and objective measurement of hearing loss (ABR and LDV measurement of RWM vibration). The highlights of this study are:

1. EH induced hearing loss progressed with the development of EH, it starts at low frequencies, and later involves medium to high frequencies.
2. EH increases the cochlear impedance, causing conductive dysfunction. This dysfunction can be cured by dehydration treatment in early stage. But irreversible sensorineural component becomes significant for long-time EH.
3. Early dehydration treatment is suggested for reserving hearing.

- This study gives an overview of the development of hearing loss in EH, and partly revealed the mechanical basis and biological influences of EH. Moreover, the investigation of dehydration treatment may provide a reference for clinical practice.

## Introduction

Menière’s disease (MD) is an inner ear disorder named after Prosper Menière (Atkinson, 1961), who in 1861 described patients with episodic vertigo accompanied by fluctuating hearing, tinnitus, and aural fullness in the affected ear. Later, the correlation between MD and endolymphatic hydrops (EH) was found by Hallpike and Cairns (1938) and Yamakawa (1938). As the presence of histopathological EH was reported postmortem, the essential relationship between EH and MD were being questioned until Merchant’s study found that EH and MD are associated with 100% of cases when the current definition of MD is strictly applied (Merchant et al., 2005; Gluth, 2020). Recently, the development of gadolinium chelate (GdC)-enhanced MRI demonstrated EH *in vivo* and further confirmed EH is a common pathological feature of inner ear diseases characterized by low-frequency hearing loss, including MD (Naganawa et al., 2014; Zou et al., 2020).

The physiological mechanisms of Méniere are still poorly understood, yet causative relationships between EH and disordered auditory physiology in MD have been supported by much clinical and experimental evidence. It is widely believed that the dysregulation of endolymph volume may lead to EH and cause a chain reaction. Since morphological changes such as the collapse of stereocilia of outer hair cells (Albers et al., 1987) or losing synapses in inner hair cells (Valenzuela et al., 2020) fail to explain the correlation of EH and hearing loss, potential explanations have mainly focused on the mechanical impact caused by high endolymphatic pressure on endolymph components (Oberman et al., 2017) and the function of the lymphatic sac (Swinburne et al., 2018). Among those theories, the mechanical mechanism of high pressure in the endolymphatic duct explains the clinical hearing symptoms in early and mid-stage Méniere’s disease (Ichimiya and Ichimiya, 2019), prompting the dehydration therapy for patients with MD. The effectiveness of dehydration therapy has not been strictly evaluated (Quaranta et al., 2019), and a recent meta-analysis further concluded that it was unclear whether diuretics were effective (Nevoux et al., 2018), even though patients have stated subjective perception improvements (Ward et al., 2019). Whether hyperosmolar agents, such as glycerol, urea, or mannitol, can improve patient hearing and act as a diagnostic tool are still under debate (Thomsen and Vesterhauge, 1979; Angelborg et al., 1982; Van de Water et al., 1986; Sterkers et al., 1987; Magliulo et al., 2001). This apparent contradiction raises the question of why patients report relief of symptoms while most of the available studies query the effectiveness of dehydration.

As the hearing ability can be predicted by the mechanical transfer of acoustic vibration and the round window is considered as an accurate proxy for cochlear fluid displacement (Ryan et al., 2020), we propose using peak-to-peak displacement of round window membrane (RWM) to analyze the change in intra-cochlear pressure under the effects of dehydration agents on EH animal models. We chose RWM as the reflection of the inner ear transfer function because it can estimate the cochlear input impedance as a whole, so it is free from the influence of the inertia of the perilymph inside the helicontrema, thus the compliance of it can better describe the total pressure inside the cochlea (Marquardt and Hensel, 2013). As the laser Doppler vibrometer (LDV) is an established optical technique to analyze the vibration of the ossicular chain, RWM, and tympanic membranes (Zhang and Gan, 2013), we used LDV to measure the dynamic behavior of RWM to evaluate cochlear impedance and sound transmission. To better illustrate the effectiveness of dehydration therapy, we created a series of different severity EH models using guinea pigs to simulate various stages of MD, then treated with mannitol. By measuring the auditory brainstem response (ABR), the linear displacement of RWM, and observing the changes in the organ of Corti, we assessed the influence of high endolymphatic pressure and the dehydration effects of mannitol on EH models.

## Materials and Methods

### Animals and Research Program

Healthy albino guinea pigs (male, weighed 250–300 g), with a positive Preyer reflex and free of tympanic membrane perforation or otitis media, were used in this study. Pre-recruiting ABR measurements (click stimuli) were conducted to exclude animals with abnormal hearing function.

Guinea pigs were classified into 10-day EH, 20-day EH, 30-day EH, and blank control groups equally (12 individuals in the blank control group and 18 animals in the EH group). For the EH group, ABR (tone burst stimuli) was conducted to evaluate the auditory responses of one-third (6 animals) of the animals, and LDV measurement was used for the other two-thirds (12 animals). For all EH groups (10, 20, and 30 days), the ABR measurements were conducted both before and after dehydration with mannitol. Bilateral ears were used in all measurements.

**FIGURE 1:**
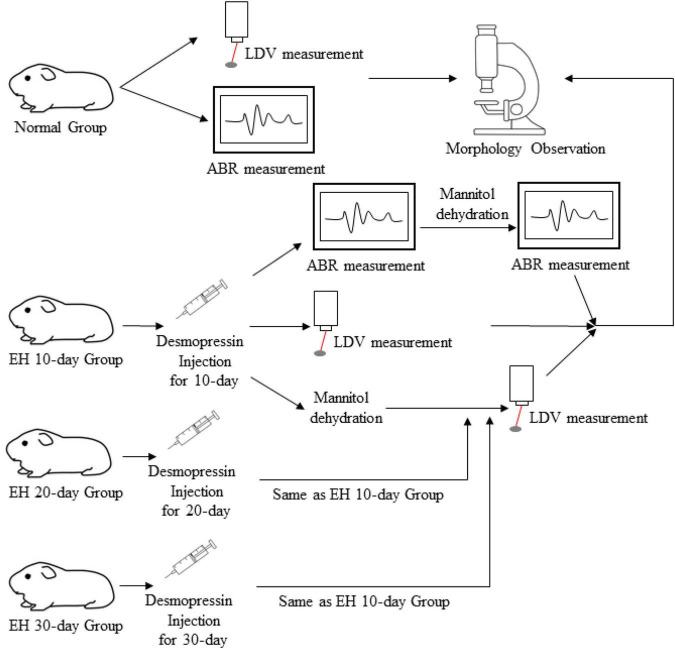
The experimental design.

For the LDV measurements, the twelve animals in each EH group were divided into two subgroups, one for direct LDV measurement and the other for vibrometry after mannitol treatment. An operation is necessary for LDV measurement, making it hard to keep the animals healthy if further mannitol treatments are followed, which may influence the effect of dehydration. The ABR and LDV results were also obtained as baselines by the blank control groups. Figure 1 and Table 1 give a flowchart and roadmap of the experimental program.

**TABLE 1:** A flowchart of the experimental procedure.

Groups	Experimental procedure		
Blank control (12 animals)	ABR (6 animals, *N* = 12)		
	LDV (6 animals, *N* = 12)		
EH 10-day (18 animals)	1st ABR (6 animals, *N* = 12)	Mannitol dehydration	2nd ABR (6 animals, *N* = 12)
	LDV (6 animals, *N* = 12)	–	–
	–	Mannitol dehydration	LDV (6 animals, *N* = 12)
EH 20-day (18 animals)	same as EH 10-day group		
EH 30-day (18 animals)	same as EH 10-day group		

After the measurements, the animals were sacrificed using an overdose of anesthetic. The cochleas were sectioned and stored in 10% polymerized formaldehyde or 2.5% glutaraldehyde for further histography.

The research program follows the principles of the guidelines for care and use of laboratory animals and was approved by the Ethics Committee of the Eye and ENT Hospital of Fudan University.

### Endolymphatic Hydrops Model and Mannitol Treatment

As several studies report the importance of the endolymphatic sac and further confirm its function of regulating endolymph (Baluk et al., 2007; Swinburne et al., 2018), we used desmopressin without combining surgery to create our EH models.

The EH guinea pig model was conducted by intraperitoneal injection of desmopressin acetate (T5144, CAS: 62288-83-9, TargetMol, United States), once a day, at a dose of 8 μg/kg weight. The dose of desmopressin reported to induce EH models was variable, but there was evidence supporting increasing dosage and injection days can provide a more severe EH status in animal models at a low dose level (Takeda et al., 2000; Katagiri et al., 2014). The dose of 4–6 μg/kg weight has been widely used for EH guinea pigs modeling (Degerman et al., 2019; Jiang et al., 2019). Considering the potential for long-time medication tolerance, we increased the dose of desmopressin for our animal models (Takeda et al., 2000). To create an EH model with different levels of severity, the duration of model creation was 10, 20, and 30 days for the 10-, 20-, and 30-day EH groups, respectively.

Different kinds of hyperosmolar dehydration agents, such as glycerol, urea, isosorbide, sodium bicarbonate, and mannitol, were used to discuss their influence on the inner ears (Baldwin et al., 1992; Yazawa et al., 1998). Given the report of the rebound phenomenon of glycerol (Takeda et al., 1999) and the recent discovery of aquaporin-3 (also termed as aquaglyceroporin) in the labyrinthine membrane (Agre et al., 2002; Beitz et al., 2003), we chose mannitol as the dehydration agent applied in this study. The percentage of mannitol used in research varies from 10 to 40% (Larsen et al., 1982; Baldwin et al., 1992; Yazawa et al., 1998; Morawski et al., 2003; Le and Blakley, 2017). A high dose of mannitol would cause a lethal effect, while a low dose may not ensure sufficient dehydration. In our experiment, the dehydration treatment was fulfilled by the intravenous injection of an 18% mannitol-saline solution at a safer dose of 0.5 g/kg. The dose and concentration were chosen by experience. During the dehydration process, the infusion rate was set slow, and the measurement started approximately an hour after the full dose of mannitol was given.

### Auditory Brainstem Responses Measurement

Before the ABR measurement, the animals were anesthetized with an intramuscular injection of ketamine hydrochloride (40 mg/kg) and xylazine hydrochloride (10 mg/kg).

The ABR measurements were conducted in a soundproof, electromagnetically shielded room. The measurements were conducted using the RZ6/BioSigRZ system (Tucker-Davis Technologies, Alachua, FL, United States). Electrodes were inserted into the skin, one electrode at the vertex for signal recording and two electrodes at the bilateral mastoids as reference and ground. Repeated tone bursts (5 ms duration, 0.5 ms rise-fall time, Blackman envelope) were presented by a closed-field speaker at a rate of 21 stimuli/s. The test frequency points are 2, 4, 8, 16, 24, and 32 kHz. The sound-intensity level ranges from 20 to 90 dB sound pressure level (SPL) at an interval of 5 dB. ABR responses were recorded and averaged after 1,024 stimuli. The threshold for each frequency point was determined typically by the ABR I, III, and V waves.

### Laser Doppler Vibrometer Measurement

The vibration of the RWM was measured to evaluate the hearing function of normal, EH-modeled, and mannitol-treated guinea pigs. The feasibility of using RWM vibration as a measure of auditory function has been previously proven (Zhang et al., 2019).

**FIGURE 2:**
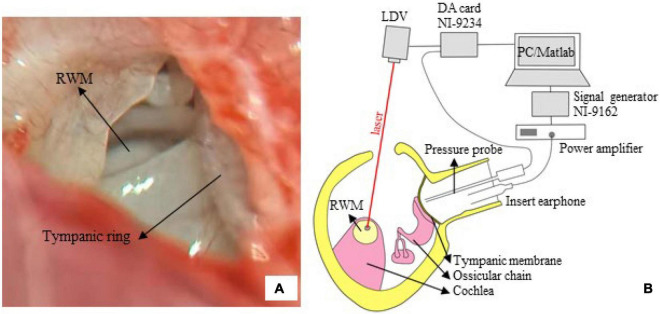
The LDV measurement. **(A)** The surgical exposure was photographed *via* a dissecting microscope. The tympanic ring and round window membrane (RWM), with a radius of 0.5–0.6 mm, are seen from the opened middle ear cavity (right ear of tested guinea pig). **(B)** A sketch of the measurement system. The RWM velocity was measured *via* a compact LDV, and the signal was acquired by an AD card (NI-9234) and analyzed by an in-house MATLAB program.

The guinea pig was anesthetized using an intramuscular injection of ketamine hydrochloride (40 mg/kg) and xylazine hydrochloride (10 mg/kg). For the duration of the surgery, the animal was placed on a heating blanket to maintain its body temperature. Bilateral auricles were partially removed, and the dorsal auditory bulla was opened to sufficiently expose the RWM (Figure 2A). A reflective tape (0.2 × 0.2 mm^2^, < 0.01 mg Polytec, Germany) was carefully placed on the center of the RWM. The tympanic membrane and the ossicular chain were kept intact.

Figure 2B illustrates the setup of the LDV system. The experiment was conducted in a soundproof chamber with < 30 dB SPL noise floor. The animal was placed on a platform. An insert earphone (ER-4PT, Etymotic, United States) was inserted into the ear canal, coupled with a tiny sound pressure probe (ER-7C, Etymotic, United States). Pure tones at 85 dB SPL in the frequency range of 0.5–10 kHz (5 points/octave) were produced by the earphones, driven by a power amplifier (Type 2718, B&K, Denmark) connected with a signal generator (NI 9263, National Instruments, United States). The tip of the pressure probe was positioned approximately 2 mm from the tympanic membrane to accurately monitor the input sound pressure.

An LDV (CLV 2534-4, Polytec, Germany) was adopted to measure the vibration of the RWM. The measuring laser beam generated by the device was controlled so that the measuring angle was greater than 70°. The RWM velocity (sensitivity: 2 mm/s/V) and input sound pressure (sensitivity: 20 Pa/V) were simultaneously recorded using a four-channel data acquisition card (NI9234, sampling rate: 51.2 kHz, National Instruments, United States). Two channels were used. An in-house MATLAB code was developed for measurement control, data acquisition, and analysis. The FFT algorithm was used to obtain the velocity amplitude and sound pressure.

### Histological Section

For histology, specimens (stored in 10% polymerized formaldehyde at 4°C) were decalcified in 10% ethylenediaminetetraacetic acid (EDTA), dehydrated with 15 and 30% sucrose solution, and coated with Optimum Cutting Temperature (OCT) compound. Then the cochlea was frozen and sectioned in the plane parallel to the modiolus (section thickness: 8 μm), followed by hematoxylin and eosin (H&E) stain.

For scanning electron microscopy (SEM), during specimen collection, the apical of the cochlea and RWM were opened, poured, and fixed with 2.5% glutaraldehyde at 4°C for 48 h. Then the membranous labyrinth was fully exposed. All other tissues were removed, leaving only the basement membrane, fixed in osmium tetroxide solution, and treated with tannic acid. Finally, the specimens were dehydrated with alcohol, dried, sprinkled with gold, and observed by a scanning electron microscope (SU8010, HITACHI, Toyko, Japan).

### Data Processing

For sections, since the EH model is induced by desmopressin, the SAM ratio is not suitable for our case. We provided changes in the angle formed by Reissner’s membrane and basilar membrane (BM) to reflect the severity of EH and response to mannitol. For LDV measured data, the RWM velocity amplitude was converted to peak-to-peak displacement according to the mathematical relation: *U*peak-*to*-peak = *V*/(π⋅*f*) (where *U*peak−*to*−peak is the peak-to-peak displacement, *V* is the velocity amplitude, and *f* is the frequency). Then the displacement is normalized by the sound pressure near the tympanic membrane. To statistically analyze the displacement and ABR threshold, the mean and standard deviation (SD) were calculated for each group. Two-tailed Student *t*-tests were used for data comparison, and *p*-values of < 0.05 were considered as a significant difference.

## Results

### Observation of Tissue Sections of the Cochlea in Ears With Endolymphatic Hydrops

**FIGURE 3:**
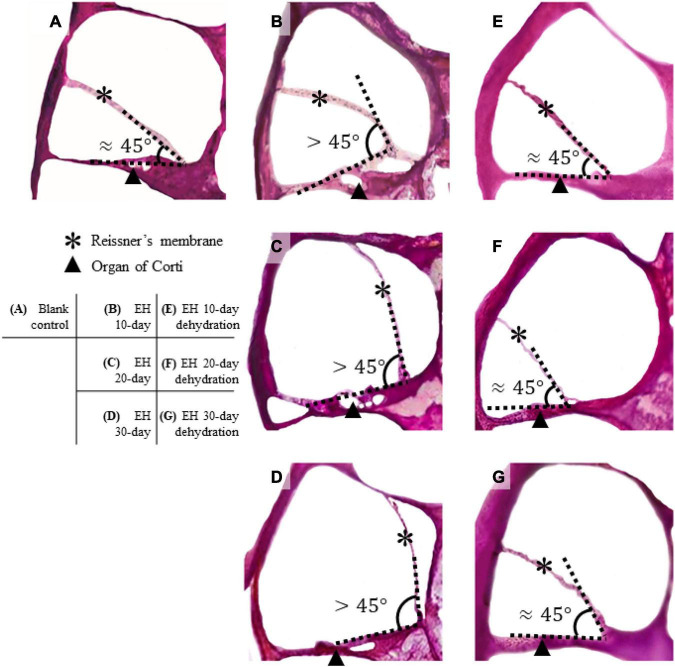
Section of cochlear turn 2 of normal, EH-modeled, and dehydration-treated guinea pigs. In the control group **(A)**, the Reissner’s membrane (RM) and the organ of Corti are in the normal position, and the RM and BM form a sharp angle (approximately 45°). In the EH groups **(B–D)**, the RM are bent, and the deformation becomes remarkable as the EH goes severe. **(E–G)** The dehydration effect where the RM is partly restored. Some distortion of the RM and the organ of Corti are shown.

Figure 3 presents the H&E stained section of the cochlea for the EH groups and the blank controlled group (more section samples are presented in Supplementary Appendix). Turn 2 was shown. In the control group (Figure 3A), the Reissner’s membrane (RM) did not deform so that the membrane and the BM formed a sharp angle. However, the RM bulged toward the scala vestibula (SV) in the EH groups (Figures 3B–D). The tissue of RM deformation became more significant with the duration of EH, indicating an increase in EH severity. These observations, consistent with the previous reports (Katagiri et al., 2014), verified our EH models.

Figures 3E–G shows turn 2 of the cochleae after dehydration. Compared with the non-dehydration results, a significant volumetric change in the scala media (SM) was shown, and in the EH 10- and 20-day groups, the RM nearly relocated to its normal location, indicating the effectiveness of mannitol treatment. Slight foldings and distortions of the RM were observed (Figures 3E–G and Supplementary Figure 1). Additionally, some cases showed the deformation of the organ of Corti (Figures 3F,G). The mean and SD of the angle between RM and BM were calculated and shown in Table 2 (sections were used to calculate the mean of the angle, thus forming the sample number of 8).

**TABLE 2:** The angle between RM and BM of turn 2 in each group (*N* = 8).

Angle between RM and BM	Mean	*SD*
Normal	41.9°	3.3
EH 10-day	59.7°	4.9
EH 20-day	77.8°	7.3
EH 30-day	90.7°	8.1
EH 10-day Mannitol Tx	50.0°	5.1
EH 20-day Mannitol Tx	54.4°	3.5
EH 30-day Mannitol Tx	57.6°	4.5

### Effect of Endolymphatic Hydrops and Dehydration Treatment on Auditory Brainstem Responses Measurement

**FIGURE 4:**
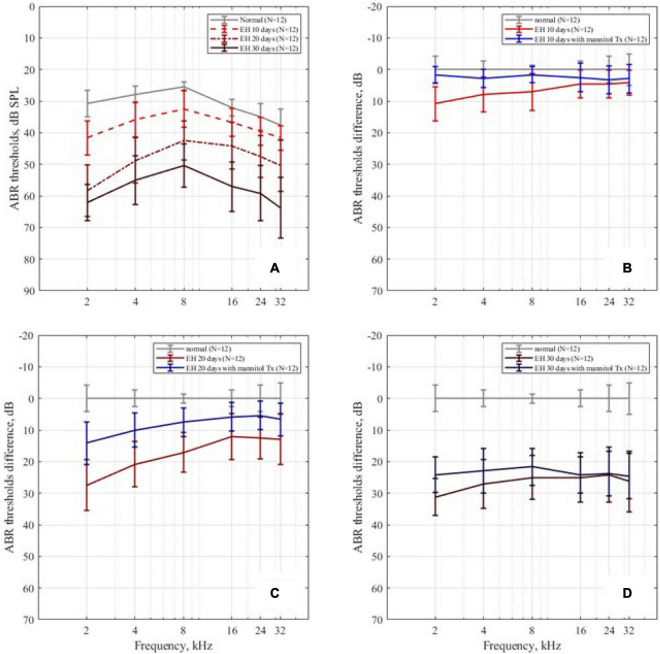
The ABR thresholds change. **(A)** The ABR thresholds in the control, EH 10-, 20-, and 30-day groups, presenting with mean and SD; as the degree of EH became more severe, the thresholds increased. **(B–D)** The ABR threshold differences between the EH groups and the dehydration groups of 10, 20, and 30 days, compared with the control group.

Auditory brainstem response thresholds of all groups are presented in Figure 4. Figure 4A plots the ABR thresholds in the EH groups of different severity, together with the result of the control group as a baseline. In the EH 10-day group, the mean thresholds increased by approximately 10 dB, mainly at low frequencies (2, 4, and 8 kHz). In the EH 20-day group, high-frequency thresholds also increased significantly (by 10–15 dB at 16, 24, and 32 kHz, *p* < 0.05), but low-frequency thresholds were still more prominent (with an increase of 20–30 dB at 2–8 kHz, *p* < 0.05). In the EH 30-day group, the ABR thresholds increased over a broad frequency range (by approximately 30 dB at 2–32 kHz, *p* < 0.05).

The mannitol dehydration treatment effect on ABR thresholds is shown in Figures 4B–D, with each sub-figure corresponding to EH 10-, 20-, and 30-day groups, respectively. Compared with non-dehydration status, the ABR thresholds at the low-frequency range of EH 10-day had remarkable improvements (9.1 dB SPL at 2 kHz, 5.0 dB SPL at 4 kHz, and 5.4 dB SPL at 8 kHz; Figure 4B, *p* < 0.05) and almost went back to normal. In the EH 20-day group, significant improvements of approximately 10 dB (*p* < 0.05) were observed in all frequencies. The improved thresholds were still notably higher than that of the normal baseline (refer to Figure 4C). In the EH 30-day group, the ABR thresholds were not changed after mannitol injection (except for a slight improvement at 2 kHz), indicating the ineffectiveness of dehydration treatment (refer to Figure 4D).

### Effect of Endolymphatic Hydrops and Dehydration Treatment on Round Window Membrane Vibration

**FIGURE 5:**
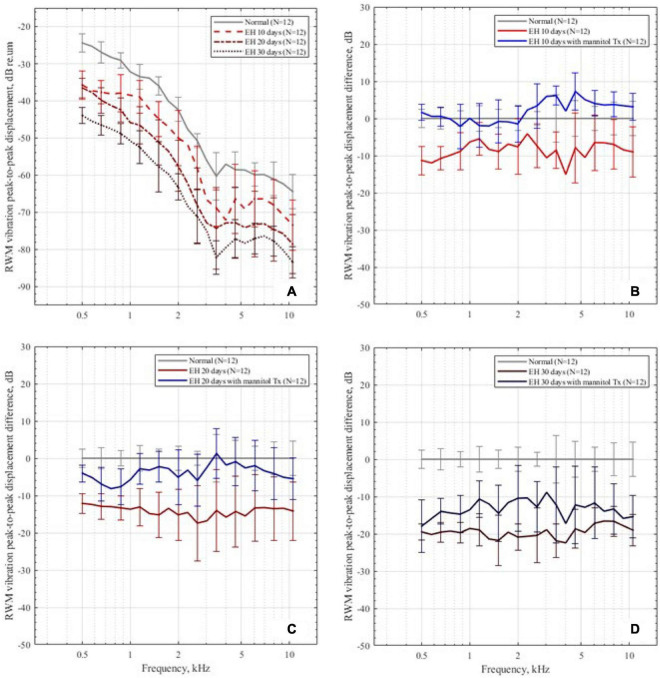
The RWM peak-to-peak displacements in the dB scale. **(A)** The RWM peak-to-peak displacement in the control group and the EH 10-, 20-, and 30-day groups, presenting with mean and SD. The displacements are normalized by sound pressure in the ear canal. **(B–D)** The RWM peak-to-peak displacement differences in the 10-, 20-, and 30-day EH groups before and after dehydration treatment, compared with the normal baseline.

Figure 5A shows the mean peak-to-peak displacement and SD of RWM in the control and EH 10-, 20-, and 30-day groups. For the normal baseline, the displacements generally decrease as frequency increases in the range of 0.5–10 kHz. The most reduction occurred at 1–5 kHz and the plateau had been reached at above 7 kHz, which coincide with the reported data (Zhang et al., 2019). In the EH 10-day group, there existed a slight reduction (approximately 10 dB) in RWM displacement at 0.5–4 kHz (11.3 dB at 0.5 kHz). For the EH 20-day group, the RWM displacement significantly decreased at the frequency range of 0.5–10 kHz (at approximately 15 dB). In the EH 30-day group, a further reduction of 15–20 dB was observed.

After dehydration treatment, the RWM vibration is shown in Figures 5B–D. In the EH 10-day group, the peak-to-peak displacement of RWM nearly went back to its normal range and was slightly higher than normal at 4–10 kHz (less than 4 dB, *p* < 0.05). In the EH 20-day group, the peak-to-peak displacements also showed a great improvement. However, at the range of 0.5–1 kHz, the recovery exhibited a mild reduction, compared with the normal curve (Figure 5C). In the EH 30-day group, the improvements were 3–8 dB, which was still 10–15 dB worse than the normal cases, suggesting the limited effect of dehydration therapy (Figure 5D).

### Observation of Outer Hair Cells of the Cochlea in Ears With Endolymphatic Hydrops

**FIGURE 6:**
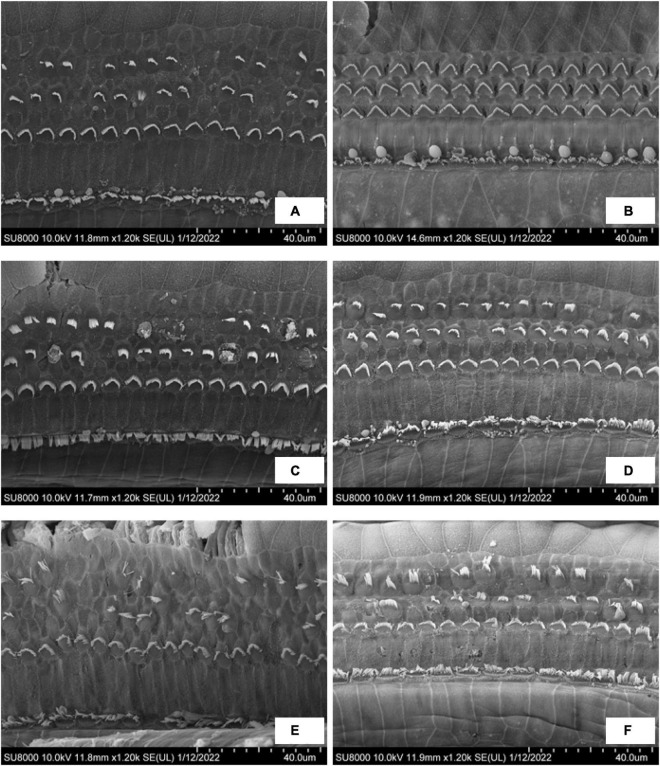
The SEM observation of OHC in the EH 10-, 20-, and 30-day groups. **(A,C,E)** Turn 2 of the cochlea in the EH 10-, 20-, and 30-day groups. **(B,D,F)** Turn 3 of the cochlea in each group accordingly.

Figure 6 shows the SEM observations of outer hair cells (OHC) in the EH 10-, 20-, and 30-day groups. Turns 2 and 3 were presented. In the EH 10-day group, the stereocilia of outer hair cells in turn 2 (Figure 6A) have already exhibited slight swelling and lodging, and accidental loss of stereocilia has been noticed. However, the OHC remains intact at turn 3 (Figure 6B). In the EH 20-day group, the damage of stereocilia of OHC in turn 2 (Figure 6C) is very obvious, and the lodging and collapse of stereocilia have started to show up in turn 3 (Figure 6D). The changes in OHC were most prominent in the EH 30-day groups. The injury to OHC was more severe compared with the EH 20-day group in both turns (Figures 6E,F), and collapse of cilia and swelling inner hair cells (IHC) have started to appear (Figures 6E,F). Those finding indicating sensation dysfunction in severe EH may be the explanation of poor response to mannitol in the EH 30-day group.

## Discussion

### The Hearing Loss in Endolymphatic Hydrops Animal Models

Animal models are commonly used in identifying and characterizing the pathophysiology of EH. Current existing animal models can resemble the dilation of SM and low-frequency hearing loss. It is widely accepted that the EH animal models, created through surgical operation, medication application, or their combination, can reproduce the chronic phase of MD (Seo and Brown, 2020).

The EH surgical model was created *via* ablation of the endolymphatic duct and endolymphatic sac by electrocauterization. It produces reliable EH models, but the permanent surgical damage and irreversible hearing loss limit its feasibility in studying the curative effect (Horner, 1991). The EH medication models are created with injections of low-dose vasopressin (VP), desmopressin, or aldosterone. In hydropic cochleae confirmed by X-ray micro-tomography (micro-CT), desmopressin influences all frequency ranges of the cochlea but is more prominent at low frequencies (Takeda et al., 2000; Chihara et al., 2013; Katagiri et al., 2014).

A similar phenomenon was observed in our EH model experiments. The ABR results showed that the hearing loss started at low frequencies (the EH 10-day group) and progressed to higher frequencies (the EH 20- and 30-day groups, refer to Figure 4A). Note that the large variations in our ABR results may be caused by individual differences in response and the vasopressin escape phenomenon (Ecelbarger et al., 1998).

The increasing hearing losses were also reflected by the reduction of RWM vibrations (refer to Figure 5A). A positive correlation was found between the reduction of the RWM vibration and the severity of EH. However, the vibration reductions were not frequency-sensitive compared with the ABR results. The RWM vibration, different from ABR thresholds, is an evaluation of the mechanical influence of EH. This may cause the inconsistency between ABR and LDV results.

### The Effect of Dehydration Therapy on Endolymphatic Hydrops

In clinical trials, dehydration agents (e.g., mannitol, isosorbide, and glycerol) are commonly used in the diagnosis and treatment of MD (Kakigi et al., 2004), as their influences on hearing improvement have been confirmed by clinical trials and research (Filipo et al., 1997; Degerman et al., 2019). The impacts of dehydration agents in animal models have usually been analyzed by tissue section or micro-CT. Among those studies, common morphological changes such as folding of the distended RM and deformation of the organ of Corti have been observed and reported (Egami et al., 2016). In our study, those morphological changes were also noticed (Figures 3F,G).

Hearing restoration after dehydration treatment was also evaluated in this study. As shown in both ABR (Figures 4B–D) and RWM vibration (Figures 5B–D) results, the hearing loss was nearly fully recovered after dehydration in the EH 10-day group and partly recovered in the EH 20-day group. In the EH 30-day group, the hearing loss was sustained. Considering the remarkable OHC injury in the EH 30-day group (Figures 6E,F), at that time, the causes of hearing loss may be transferred from conductive dysfunction to sensory impair, restoring of conductive function by dehydration is not enough to improve hearing.

### Conductive and Sensorineural Hearing Loss

Typically, patients with MD experience fluctuating low-frequency hearing loss followed by medium- to high-frequency involvement. Gluth (2020) reviewed the hypothesis for MD development. The mechanical effect of high endolymphatic pressure affects cochlear conductive problems and damages the sensory hair cells, resulting in sensation dysfunction. The same damage to hair cell stereocilia in our study was also reported in many EH animal models (Momin et al., 2010; Jia et al., 2012; Ding et al., 2016).

Our results support this assumption. In the EH 10-day group, the injury stereocilia of OHC at turn 2 is minimal, and turn 3 is almost normal. The RWM vibration decreased by 7.6, 15.0, and 7.0 dB at 2,4, and 8 kHz, respectively (referring to normal range in Figure 5B), which were similar to ABR threshold changes (10.8, 7.9, and 7.1 dB at 2, 4, and 8 kHz, respectively, refer to Figure 4B). In the EH 20-day group, the hearing loss evaluated by RWM vibration was 15.2, 15.8, and 13.5 dB at 2, 4, and 8 kHz, respectively (Figure 5C), while the ABR threshold elevation was significantly higher (27.5, 20.8, and 17.1 dB at 2, 4, and 8 kHz, respectively, refer to Figure 4C). The gap was even more significant in the EH 30-day group. The ABR increases were 31.3, 27.1, and 25.0 dB, and the LDV results were 20.9, 22.4, and 16.6 dB at 2, 4, and 8 kHz, respectively (Figures 4D, 5D). For long-time EH models, the hearing loss evaluated by ABR was higher than that by RWM vibration, which is consistent with OHC stereocilia injury (Figures 6C–F).

The RWM vibration is a measurement of cochlear impedance, representing the conductive dysfunction component in acoustic transferring, while the ABR change is a sum of the conductive and sensation dysfunction. Therefore, the gap between ABR and RWM vibration may represent the SNHL. In our study, there is no gap in the EH 10-day animal models, indicating minimal SNHL at that time. However, the gap occurs in the EH 20- and 30-day groups, suggesting an increasing sensorineural component. Therefore, the dehydration treatment shows a significant curative effect in the EH 10- and 20-day groups (Figures 3B, 4B) while less effective in the EH 30-day group (Figures 3C,D, 4C,D).

### Endolymphatic Hydrops-Induced Cochlear Damage

Besides the dislocations of RM in EH, the BM may also deform (Xenellis et al., 2004) as shown in Figure 3, caused by the pressure difference between SM and scale tympani. However, the BM deformation is quite small, and sectioning observations may not be solid evidence of BM deformation since slight deformation may also be caused during the sectioning. The BM deformation in EH has been numerically calculated using finite element analysis (Lee and Koike, 2017; Areias et al., 2021). The numerical analysis also predicts a low-frequency conductive hearing loss caused by BM deformation.

There are also assumptions that the EH-induced BM deformation may damage the sensory cells and cause SNHL. Lee and Kalinec (2016) measured the deformation of the organ of Corti in acute guinea pig EH models. They observed a significant decrease in the average area and height of the organ of Corti in the apical turn. They found that the lengths of OHC and Deiters’ cells in the apical turn were significantly reduced. Lee suggested that the compression and deformation of the organ of Corti may decouple the tectorial membrane and stereocilia. However, *in vivo* observations need further solid evidence. Optical coherence tomography may be a promising technique (Liu et al., 2017; Badash et al., 2021).

### Limitations of This Study

The first limitation of this study is the mismatch between the frequency range in ABR and LDV measurements. The LDV measurement range was 0.5–10 kHz. Frequencies beyond this interval had not been surveyed, so the paired frequencies in ABR thresholds and RWM vibrations were restricted to 2–8 kHz. Second, mannitol injection is used in the diagnosis of MD in clinical practice instead of treatment due to severe side effects. However, it does not affect the conclusion of this study.

## Conclusion

This study investigated the development of hearing loss in guinea pig EH models as well as the effect of dehydration treatment. By combining analogical observations (frozen section) and objective measurements of hearing loss (ABR and LDV measurement of RWM vibration), we are able to obtain an overview of the development of hearing loss in EH, both mechanically and biologically. The main conclusions of this study are as follows:

1. EH-induced hearing loss progressed with the development of EH; it starts at low frequencies and later involves medium to high frequencies.
2. EH increases the cochlear impedance, causing conductive dysfunction. This dysfunction can be cured by dehydration treatment at an early stage. But the irreversible sensorineural component becomes significant for long-time EH.
3. Early dehydration treatment is suggested for preserving hearing.

## Data Availability Statement

The original contributions presented in the study are included in the article/Supplementary Material, further inquiries can be directed to the corresponding author/s.

## Ethics Statement

The animal study was reviewed and approved by institutional animal care and use committee at Eye and ENT Hospital of Fudan University.

## Author Contributions

S-QW: methodology, formal analysis, visualization, and writing—original draft. C-LL: methodology, formal analysis, visualization, and writing—review and editing. J-QX and L-LC: methodology. Y-ZX and P-DD: writing—review and editing. L-JR: conceptualization, software, writing—review and editing, supervision, and funding acquisition. W-JY: supervision and funding acquisition. T-YZ: conceptualization, supervision, and funding acquisition. All authors contributed to the article and approved the submitted version.

## Conflict of Interest

The authors declare that the research was conducted in the absence of any commercial or financial relationships that could be construed as a potential conflict of interest.

## Publisher’s Note

All claims expressed in this article are solely those of the authors and do not necessarily represent those of their affiliated organizations, or those of the publisher, the editors and the reviewers. Any product that may be evaluated in this article, or claim that may be made by its manufacturer, is not guaranteed or endorsed by the publisher.
