# Supplementary material for: The Effect of Endolymphatic Hydrops and Mannitol Dehydration Treatment on Guinea Pigs
Source: Front Cell Neurosci. 2022 Apr 11;16:836093. doi: 10.3389/fncel.2022.836093 (PMC9035551; doi:10.3389/fncel.2022.836093)
Supplement: Supplementary file 1 [file Image_1.pdf]

Appendix

Here we provide other four cochlea sections of each group. Individual response to desmopressin and mannitol is various, but the trend of increasing endolymphatic hydrops (EH) severity with injection days is obvious and mannitol dehydration works.

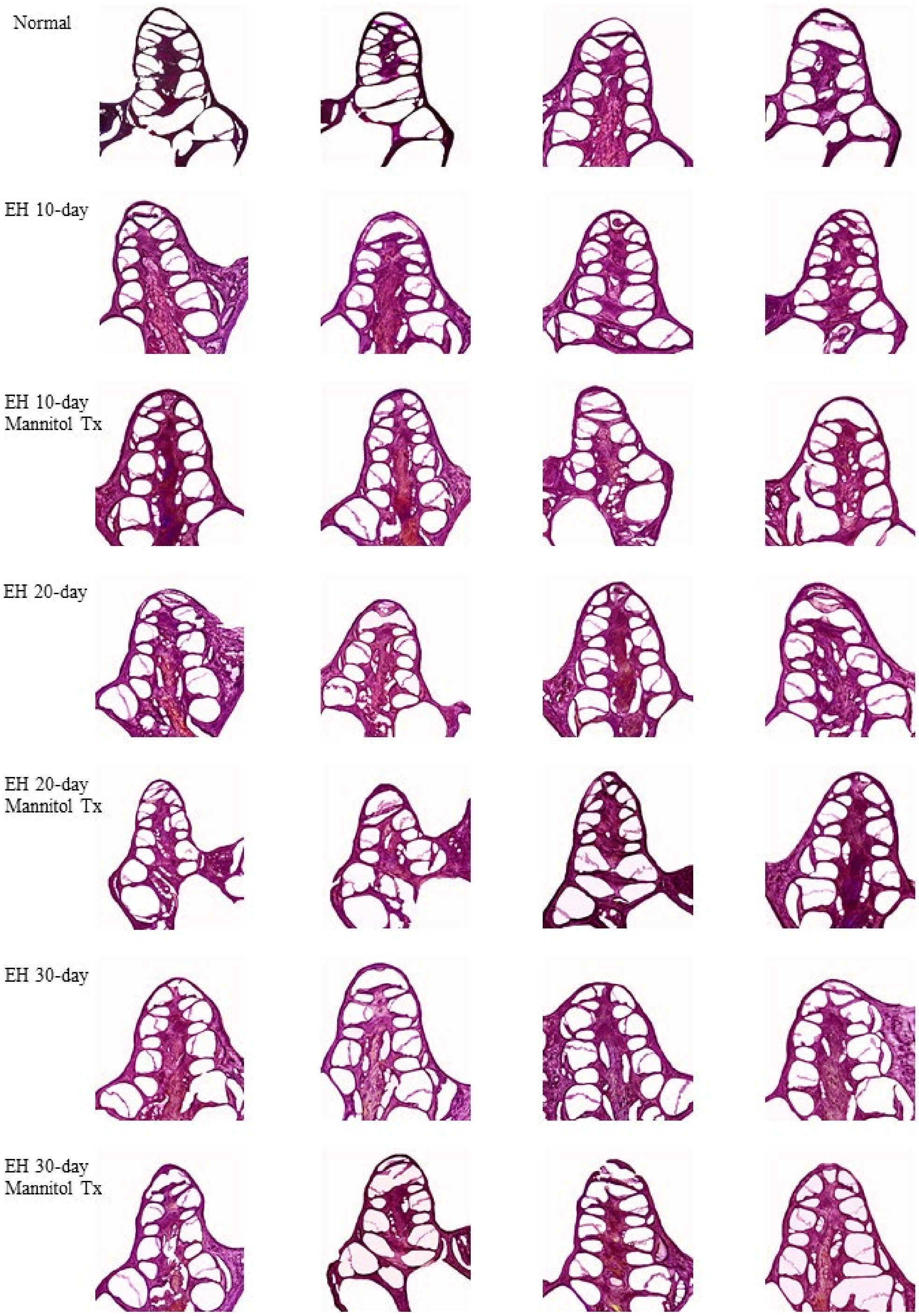

Fig.S1 Sections of cochlea in normal, EH 10-, 20-, 30-day groups and responses to mannitol treatment. Noticed that shrinkage of Reissner's membrane (RM) is remarkable at dehydration groups.
